# Primitive neuronal component is a frequent finding among IDH-mutant astrocytomas with *RB1* alterations

**DOI:** 10.1007/s00401-026-03014-5

**Published:** 2026-04-19

**Authors:** Cheyanne C. Slocum, Jamal K. Benhamida, Scott Eckert, Erika Gedvilaite, Ryan Ptashkin, Viviane Tabar, Robert J. Young, Marc Ladanyi, Ingo K. Mellinghoff, Marc K. Rosenblum, A. Rose Brannon, Richard Hickman, Tejus A. Bale

**Affiliations:** 1 Deparment of Pathology, New York Presbyterian Hospital, NY, New York USA; 2 Department of Pathology and Laboratory Medicine, Memorial Sloan Kettering Cancer Center, New York, USA; 3 Department of Neuro-Oncology, Memorial Sloan Kettering Cancer Center, New York, NY USA; 4 Deparment of Neurosurgery, Memorial Sloan Kettering Cancer Center, New York, NY USA; 5 Department of Radiology, Memorial Sloan Kettering Cancer Center, New York, NY USA; 6 Department of Pathology, Icahn School of Medicine at Mount Sinai, New York, NY USA; 7 Natera, TX, Austin USA; 8 Foundation Medicine, Inc., Cambridge, MA, USA

Molecular biomarkers for risk stratification among IDH-mutant astrocytomas have taken precedence over histologic grading. Meanwhile, although predominant morphologic patterns and histologic subtypes are well-documented, the morphology is thought to reflect diverse tumor cell states, and the clinical relevance remains unclear [1, 6–8, 12]. Improved understanding requires histologic re-examination of well-annotated tumors with relatively uniform genomic backgrounds. Primitive neuronal morphology in both glioblastoma, IDH-wildtype as well as IDH-mutant astrocytomas has recently been associated with a characteristic molecular signature, including a unique methylation profile [4, 10]. While rare, given the reported aggressive nature of these tumors, including frequent leptomeningeal dissemination and outcomes equivalent to grade 4 tumors, the associated molecular features require further study [4, 5, 13]. Loss of RB1 function/expression and the acquisition of a “small cell” neuroendocrine phenotype are progression-related phenomena potentially displayed by a variety of solid tumors [2, 9, 11]. Further, RB1 loss in and of itself has been reported as a negative prognostic indicator in IDH-mutant astrocytoma, although to date homozygous deletion of *CDKN2A/2B* remains the only *RB1* pathway alteration included in WHO grading criteria [1, 7]. Therefore, we sought to understand the implications of primitive neuronal component (PNC) in astrocytoma specifically within the genomic context of *IDH1* and *RB1* co-mutation.

From 463 previously reported IDH-mutant gliomas, we identified 25 IDH-mutant astrocytomas (10 recurrent, 15 newly diagnosed) with deleterious alterations in *RB1* for histologic review, molecular characterization, and review of records [3]. Research review of a corresponding H&E section was performed to ensure that the primitive neuronal component constituted > 50% of the evaluable tumor in each case. Among *IDH1/RB1* co-mutant astrocytomas, 52% (13/25) of the cases had a well-defined PNC (6 at recurrence, 7 at diagnosis), characterized by high nuclear/cytoplasmic ratio, dense cellularity, and brisk mitotic activity (Fig. 1A-C). All tumors with PNC met 2021 histologic criteria for CNS WHO grade 4 (1/13 tumors also demonstrated homozygous deletion of CDKN2A/2B), as did most tumors without PNC (75%, 9/12, Fig. 1D). Grade 4 tumors with *RB1* alterations demonstrated comparable overall survival to other Grade 4 IDH-mutant astrocytomas ( HR: 0.82 (CI: 0.44–1.56), p = 0.6). Immunohistochemical characterization of the primitive component was performed in 12/13 cases, which supported neuronal differentiation as evidenced by expression of synaptophysin (12/12) with at least partial loss of GFAP (9/12) and frequent loss of RB expression in most cases (8/12).

**Fig. 1:**
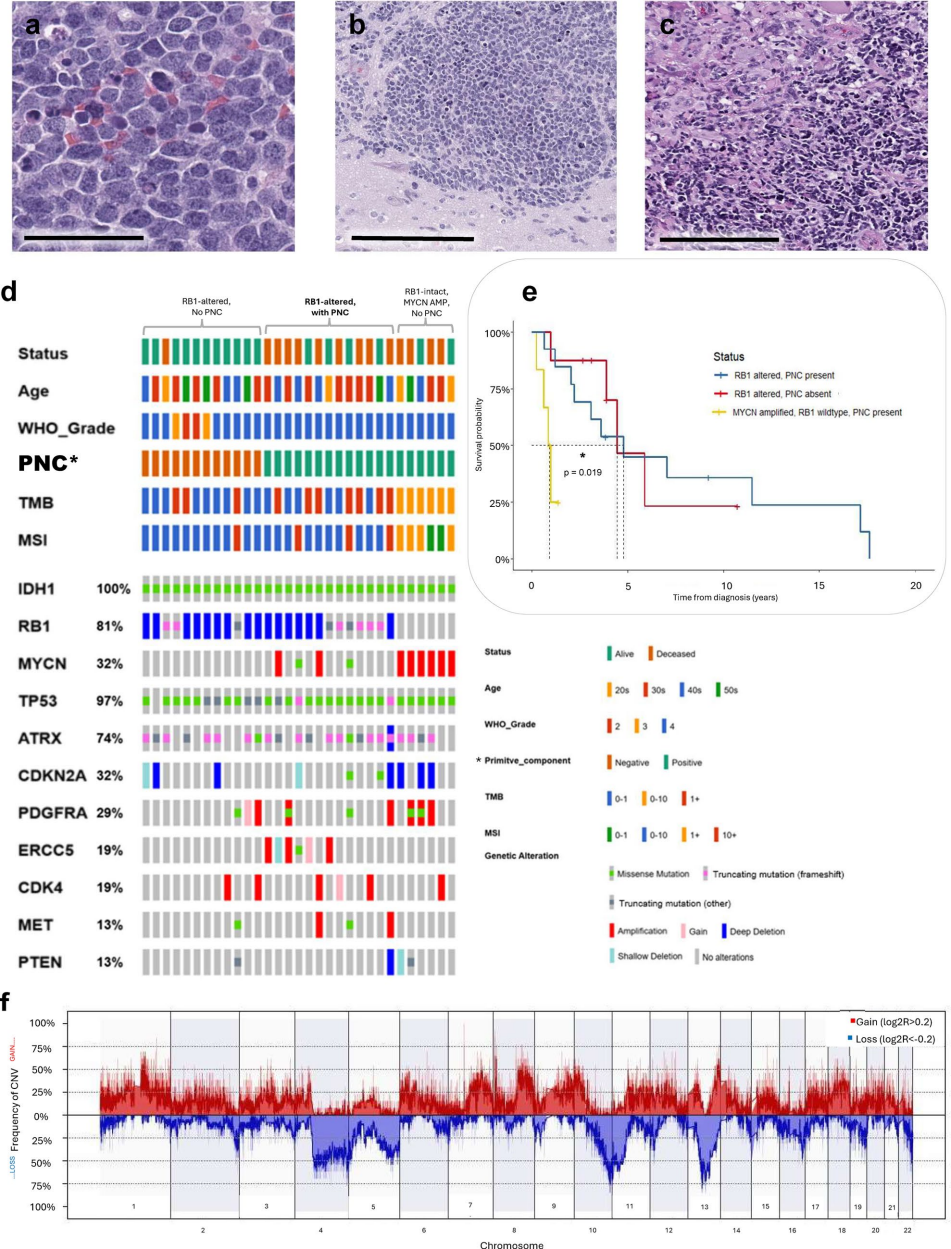
(**A**–**C**) Representative images of primitive neuronal component (PNC) in IDH-mutant astrocytoma. Comprised of a dense proliferation of small blue cells with frequent cell wrapping, nuclear molding, and brisk mitotic activity (**A**), PNC may present as the predominant morphologic pattern (**B**) or co-exist with astrocytic-appearing tumor (**C**, Scale bars: A = 50 μm, B = 200 μm, C = 200 μm). **D** Oncoplot and cohort characteristics of 25 IDH1/RB1 co-mutant astrocytomas, 52% (13/25) of which demonstrated PNC. **E** PNC was not associated with significantly shorter overall survival among grade 4 *IDH1/RB1* co-mutant astrocytomas (p = 0.89. HR: 1.09, 95% CI: 0.32- 3.67), however, *MYCN*-amplified tumors with PNC showed significantly shorter survival (HR: 8.33, 95% CI 1.43- 50.0, P = 0.019. **F** Cumulative copy number variant frequency of *IDH1/RB1* co-mutant astrocytomas with PNC derived from methylation profiling data (n = 13)

IDH-mutant astrocytomas with PNC demonstrate an aggressive disease course with median overall survival of 57.2 months, varying from 7.6 to 211 months after initial diagnosis. However, among grade 4 *IDH1/RB1* co-mutant astrocytomas, the presence of a PNC was not associated with significantly shorter overall survival (p = 0.89, hazard ratio [HR]: 1.09, 95% CI: 0.32- 3.67, Fig. 1E). While no tumors in our cohort with available MRI imaging demonstrated leptomeningeal disease, the incidence of subependymal and/or subpial/perivascular disease was frequent, though not significantly different among tumors with or without PNC (38% (5/13) v. 55% (6/11); p = 0.4528).

Tumors with PNC did not demonstrate significantly increased tumor mutation burden (TMB, mean 23.4 v. 4.87, p = 0.19), microsatellite instability (MSI, mean 1.32 v. 0.89, p = 0.47), or chromosomal instability as reflected by higher copy number alterations (CIN, mean 0.081 v. 0.093, p = 0.5, Fig. 1E and supplemental), by targeted hybrid capture next-generation sequencing (341–505 cancer genes, MSK-IMPACT). 100% of cases with PNC also demonstrated mutations in *TP53* (13/13) while only 7.7% (1/13) had a mutation in *PTEN*. Additional recurrent alterations included *ATRX* mutations (84.6%, 11/13), *ERCC5* gain (30.8%, 4/13), *CDKN2A/2B* loss (23.1%, 3/13) and focal amplifications of *CDK4* (23.1%, 3/13), *MET* (15.4%, 2/13), *PDGFRA* (15.4%, 2/13), and *MYCN* (15.4%, 2/13, Fig. 1E). DNA methylation profiling demonstrated a high-confidence match to methylation class (MC) “MC Astrocytoma, IDH-mutant; high grade” in 11/13 tumors (mean calibrated score (CS): 1.00). One tumor returned “MC Astrocytoma, IDH-mutant; lower grade” with low probability (CS = 0.57). The remaining tumor returned no valid classifications above CS = 50. (Supplemental Table 1).

We conclude that a PNC is a frequent phenotype among *IDH1/RB1* co-mutant tumors, which display other grade 4 features. Our findings suggest that many of the aggressive clinical features attributed to PNC may be inherent to unfavorable molecular profiles, (including *RB1* alterations). We recognize that PNC is not a phenotype unique to *RB1*-altered tumors and has been previously reported in association with alterations in several genes, many of which co-occur with *RB1* alterations (Fig. 1D). In our cohort of IDH-mutant astrocytomas, we identified additional tumors with intact *RB1* and with amplifications in *MET* (n = 6), *CDK4* (n = 11), *PDGFRA* (n = 7), and *MYCN* (n = 8). Among these, a PNC was identified in 8 tumors, 6 of which demonstrated *MYCN* amplification (the remaining 2 showed *CDK4* amplification). As these 6 WHO grade 4 tumors (*IDH1*-mutant/ *RB1*-intact/*MYCN-* amplified with PNC) reflected a relatively uniform group, we compared these tumors to *IDH1*-mutant, *RB1*-mutant tumors with PNC.

While we find that almost all tumors among all three groups (*IDH1/RB1*-mutant without PNC, *IDH1/RB1* mut with PNC, *IDH1*-mutant/*RB1*-intact/*MYCN*-amplified with PNC) demonstrate high confidence matches to “MC Astrocytoma, IDH-mutant; high grade” by DNA methylation profiling (Supplemental Table 1), hierarchical clustering distinguishes tumors with PNC from those without, supporting prior reports that IDH-mutant astrocytomas with PNC have a distinct methylation profile (Supplemental Fig. 1). However, the *MYCN*-amplified with PNC group showed significantly shorter survival (Fig. 1E, HR: 8.33, 95% CI 1.43- 50.0, P = 0.019), suggesting that specific molecular alterations may allow for further risk stratification among tumors with PNC.

Our findings highlight the complementary contribution of histologic review, broad sequencing and methylation profiling to tumor classification. However further study is needed; higher resolution techniques, including transcriptome or single-cell based analyses, applied to larger sample cohorts may help to uncover the mechanisms mediating this aggressive growth pattern. Future studies may benefit from microdissection of the PNC, which was not performed here. The true prevalence of PNC among *IDH1*-mutant astrocytomas is difficult to assess from large retrospective cohorts, as reporting of morphologic phenotypes has not been standardized; ongoing questions addressing the relevance of morphologic patterns in glioma may also be aided by the advancement of digital pathology. Ultimately however, the success of future investigations remains dependent on the continued efforts of neuropathologists to recognize and annotate unusual morphologic patterns among gliomas and, as our findings underscore, performing analyses within uniform molecular contexts.

## Supplementary Information

Below is the link to the electronic supplementary material.

Supplementary file1 (PNG 244 KB)

Supplementary file2 (XLSX 13 KB)

Supplementary file3 (DOCX 62 KB)

## Data Availability

The datasets analyzed in the current study are available from the corresponding author on reasonable request.The datasets analyzed in the current study are available from the corresponding author on reasonable request.
