# Supplementary figures and images for: Primitive neuronal component is a frequent finding among IDH-mutant astrocytomas with RB1 alterations
Source: Acta Neuropathol. 2026 Apr 19;151(1):42. doi: 10.1007/s00401-026-03014-5 (PMC13092521; doi:10.1007/s00401-026-03014-5)

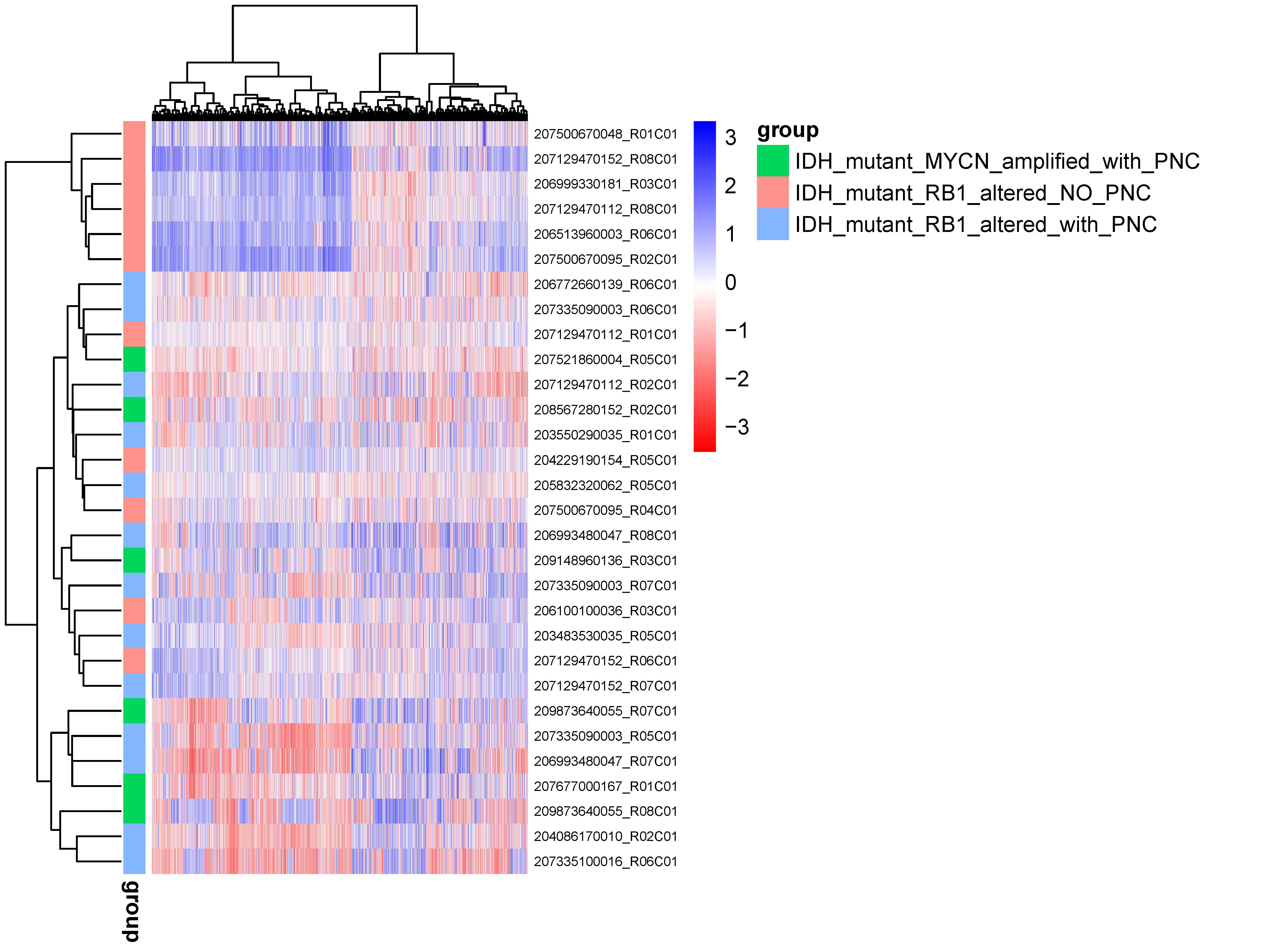

Supplement: Supplementary file 1 — Supplementary file1 (PNG 244 KB) [file 401_2026_3014_MOESM1_ESM.png]
