# Supplementary material for: Primitive neuronal component is a frequent finding among IDH-mutant astrocytomas with RB1 alterations
Source: Acta Neuropathol. 2026 Apr 19;151(1):42. doi: 10.1007/s00401-026-03014-5 (PMC13092521; doi:10.1007/s00401-026-03014-5)
Supplement: Supplementary file 3 — Supplementary file3 (DOCX 62 KB) [file 401_2026_3014_MOESM3_ESM.docx]

**Acknowledgements**

This work was partly funded by the Marie-Josée and Henry R. Kravis Center for Molecular Oncology and the National Cancer Institute Cancer Center Core Grant No. P30-CA008748. The authors thank Shadia Carlo for administrative assistance in preparation of this article, as well as the members of the Molecular Diagnostics Service in the MSK Department of Pathology and Laboratory Medicine.

**Supplementary methods**

**Ethical approval**

This study was conducted in accordance with the Declaration of Helsinki and was approved by the Institutional Review Board at Memorial Sloan Kettering Cancer Center. This protocol allows for clinicopathologic and molecular data retrieval from patients who provided informed consent or a waiver of consent.

**Cohort Assembly**

The MSK-IMPACT™ database was retrospectively searched for astrocytomas with *IDH1* or *IDH2* mutations as previously described [6]. In short, from 463 tumor samples retrieved, 347 samples remained after exclusion of non-hotspot *IDH1*/*IDH2* mutations, cerebrospinal fluid samples, and detection of 1p/19q codeletion by copy number analysis or fluorescence in-situ hybridization. Samples included both newly diagnosed and recurrent tumors. From this cohort, 25 IDH-mutant astrocytomas with deleterious alterations in *RB1* were identified for histologic review by at least two board-certified pathologists subspecialized in neuropathology (MKR, RH, TAB) with confirmation of the molecular classification performed by pathologist with additional board-certification in molecular genetic pathology (RH, TAB). Additional clinicopathologic information was retrieved from electronic medical records.

**Histopathology**

All available slides were retrieved and were rereviewed for diagnostic confirmation and assessment of histologic and immunohistochemical features. Formalin-fixed and paraffin-embedded (FFPE) tissues were utilized for all immunohistochemical and molecular diagnostic assays, which were performed in the course of clinical care. The streptavidin–biotin peroxidase complex method was utilized for immunohistochemical studies as described, with antibodies as follows: synaptophysin (SYN; Biogenex, monoclonal), glial fibrillary acidic protein (GFAP; Cell Marque, monoclonal), retinoblastoma (RB, Leica, monoclonal).

Prior to molecular testing, a representative hematoxylin and eosin-stained slide was examined to assess for estimated tumor purity >10% for sequencing . Research review of these sections was performed for the purpose of this study to ensure that the primitive neuronal component constituted > 50% of the evaluable tumor.

**Genetic analysis**

***Next generation sequencing (NGS)***

Sequencing was performed as previously described by MSK-IMPACT™, an FDA-authorized hybridization capture-based targeted NGS assay [5]. Briefly, the assay employs custom capture probes that target all protein-coding exons and select intronic regions of 341 to 505 cancer-related genes, for somatic variant detection by tumor-matched normal sequencing and analyzed by clinically validated bioinformatic pipeline: captured libraries were sequenced on Illumina HiSeq 2500 or NovaSeq 6000 instruments. Point mutations and indels were detected using MuTect, Vardict and SomaticIndelDetector and Structural variants (SV) were identified using DELLY [10]. Coverage-based copy number alterations were detected by comparing loess-normalized sequence coverage of targeted loci of the tumor with a standard diploid non-tumor sample, and annotated using VEP/ ANNOVAR. Tumor mutational burden (TMB) was calculated as nonsynonymous coding mutations per megabase (Mb). Fraction of genome altered (FGA), defined by proportion of the genome affected by copy number gains or losses, was downloaded for each sample using cBioPortal [4]. Microsatellite instability (MSI) assessment by MSIsensor: MSIsensor Methodology: Microsatellite instability (MSI) status is assessed using the MSIsensor program [8]. All variants were reviewed and called by a bioinformatic analyst and a board-certified molecular genetic pathologist.

Allele-specific copy number analysis was performed using FACETS [11]. Allelic imbalance (loss of heterozygosity (LOH)) was assessed using the allele log-odds-ratio (logOR), which compares the variant allele read count of heterozygous single nucleotide polymorphism (SNP) sites in the tumor with that in the normal tissue. The diploid state (diplogR) was manually reviewed by a bioinformatician and optimized according to diploid chromosomal states (total copy number (tcn) = 2, lesser/ minor copy number (lcn) = 1).

Homozygous deletion (HOMDEL) of *RB1* is defined as tcn:lcn 0:0, hemizygous deletion (HEMIDEL) is defined as tcn:lcn 1:0, while copy neutral loss of heterozygosity (CNLOH) refers to tcn:lcn 2:0 in *RB1*.

*RB1* alterations were considered deleterious if at least one of the following were identified: pathogenic or likely pathogenic variants including missense, frameshift, nonsense or splice site (as determined by OncoKb (PMID: 28890946), deep deletion by coverage-based copy number analysis with confirmation of homozygous deletion by allele-specific copy number analysis.

**Chromosomal instability (CIN) score algorithm**

Integer copy number analysis from the FACETS2n [9] algorithm were generated for each tumor-normal sample pair. Scores were calculated using a stepwise algorithm on a single pass through the genome, following a similar procedure to a prior CIN calculation method [7]. Rather than raw magnitude of the events in their calculation, this approach favors gains and amplifications over losses. We adjusted the method to score the magnitude of amplification (total copy number – ploidy > 4) events to be equal to that of homozygous deletions, and gains to be equivalent to heterozygous losses. The FACETS algorithm [11] allows for detection of copy-neutral loss of heterozygosity (CNLOH). Relative to a balanced diploid locus, CNLOH was scored as two events. The score was standardized to the number of target genes on the panel. Sex chromosomes were excluded from the analysis.

**Genome-wide DNA methylation profiling**

Corresponding H&E sections from each sample were reviewed to ensure that the majority of tumor cells demonstrated primitive neuronal morphology. Methylation profiling was perform as previously described using the Infinium MethylationEPIC (850K) platform [2, 3]. Briefly, 50- 250 ng of input genomic DNA, extracted from FFPE tissue (Chemagic DNA Tissue kit, PerkinElmer chemagen Technologie, GmbH, Baesweiler, Germany), were bisulfite converted (EZ DNA Methylation Kit; Zymo Research; catalog number D5002), followed by FFPE restoration step (Infinium HD FFPE DNA Restore Kit; Illumina; catalog number WG-321-1002). Samples were processed and scanned according to manufacturer's recommended protocol using the Infinium 850k array and Illumina iScan. Cases were assigned methylation-based classes using version 12.8 of the brain classifier obtained from the German Cancer Research Center, as previously described (DKFZ). Methylation class matches are defined by calibrated scores above 0.7 (inclusive). Qualified methylation matches are defined as calibrated scores greater than 0.5 (inclusive) and less than 0.7. Methylation classes and MCFs are not reported if they are less than 0.5. [1, 3].

Hierarchical clustering was performed on the same 10,000 CpG loci using the R pheatmap package using the Ward D2 method.

Copy number profiles were generated using the R Conumee package. Cumulative CNV frequencies were derived by extracting bin-level log ratios from the conumee cnv data and defining gain and loss thresholds as 0.2 and -0.2, respectively, for frequency calculations.

**Statistical analysis**

Follow-up time for a sample/patient was defined as the duration of time from surgical procedure until death (event) or last follow-up (right censored). Overall survival (OS) was calculated using the Kaplan-Meier method using OriginPro (Microcal). The log-rank test was used to assess significant differences in OS across defined groups. Hazard Ratio with corresponding 95% confidence intervals (CI) was calculated in Microsoft Excel.

1 Benhamida JK, Harmsen HJ, Ma D, William CM, Li BK, Villafania L, Sukhadia P, Mullaney KA, Dewan MC, Vakiani Eet al (2023) Recurrent TRAK1::RAF1 Fusions in pediatric low-grade gliomas. Brain Pathol 33: e13185 Doi 10.1111/bpa.13185

2 Benhamida JK, Hechtman JF, Nafa K, Villafania L, Sadowska J, Wang J, Wong D, Zehir A, Zhang L, Bale Tet al (2020) Reliable Clinical MLH1 Promoter Hypermethylation Assessment Using a High-Throughput Genome-Wide Methylation Array Platform. J Mol Diagn 22: 368-375 Doi 10.1016/j.jmoldx.2019.11.005

3 Capper D, Jones DTW, Sill M, Hovestadt V, Schrimpf D, Sturm D, Koelsche C, Sahm F, Chavez L, Reuss DEet al (2018) DNA methylation-based classification of central nervous system tumours. Nature 555: 469-474 Doi 10.1038/nature26000

4 Cerami E, Gao J, Dogrusoz U, Gross BE, Sumer SO, Aksoy BA, Jacobsen A, Byrne CJ, Heuer ML, Larsson Eet al (2012) The cBio cancer genomics portal: an open platform for exploring multidimensional cancer genomics data. Cancer Discov 2: 401-404 Doi 10.1158/2159-8290.CD-12-0095

5 Cheng DT, Mitchell TN, Zehir A, Shah RH, Benayed R, Syed A, Chandramohan R, Liu ZY, Won HH, Scott SNet al (2015) Memorial Sloan Kettering-Integrated Mutation Profiling of Actionable Cancer Targets (MSK-IMPACT): A Hybridization Capture-Based Next-Generation Sequencing Clinical Assay for Solid Tumor Molecular Oncology. J Mol Diagn 17: 251-264 Doi 10.1016/j.jmoldx.2014.12.006

6 Hickman RA, Gedvilaite E, Ptashkin R, Reiner AS, Cimera R, Nandakumar S, Price A, Vanderbilt C, Fahy T, Young RJet al (2023) CDKN2A/B mutations and allele-specific alterations stratify survival outcomes in IDH-mutant astrocytomas. Acta Neuropathol 146: 845-847 Doi 10.1007/s00401-023-02639-0

7 Hortobagyi GN, Chen D, Piccart M, Rugo HS, Burris HA, 3rd, Pritchard KI, Campone M, Noguchi S, Perez AT, Deleu Iet al (2016) Correlative Analysis of Genetic Alterations and Everolimus Benefit in Hormone Receptor-Positive, Human Epidermal Growth Factor Receptor 2-Negative Advanced Breast Cancer: Results From BOLERO-2. J Clin Oncol 34: 419-426 Doi 10.1200/JCO.2014.60.1971

8 Niu B, Ye K, Zhang Q, Lu C, Xie M, McLellan MD, Wendl MC, Ding L (2014) MSIsensor: microsatellite instability detection using paired tumor-normal sequence data. Bioinformatics 30: 1015-1016 Doi 10.1093/bioinformatics/btt755

9 Ptashkin RN, Ewalt MD, Jayakumaran G, Kiecka I, Bowman AS, Yao J, Casanova J, Lin YD, Petrova-Drus K, Mohanty ASet al (2023) Enhanced clinical assessment of hematologic malignancies through routine paired tumor and normal sequencing. Nat Commun 14: 6895 Doi 10.1038/s41467-023-42585-9

10 Rausch T, Zichner T, Schlattl A, Stutz AM, Benes V, Korbel JO (2012) DELLY: structural variant discovery by integrated paired-end and split-read analysis. Bioinformatics 28: i333-i339 Doi 10.1093/bioinformatics/bts378

11 Shen R, Seshan VE (2016) FACETS: allele-specific copy number and clonal heterogeneity analysis tool for high-throughput DNA sequencing. Nucleic Acids Res 44: e131 Doi 10.1093/nar/gkw520
